# A transition of ω-Fe_3_C → ω′-Fe_3_C → θ′-Fe_3_C in Fe-C martensite

**DOI:** 10.1038/s41598-020-63012-9

**Published:** 2020-04-08

**Authors:** D. H. Ping, H. P. Xiang, H. Chen, L. L. Guo, K. Gao, X. Lu

**Affiliations:** 1 National Institute for Materials Science, sengen 1-2-1, Tsukuba, 305-0047 Japan; 2 School of Materials Science and Engineering, Tongji University, Shanghai, 201804 China; 3 School of Materials Science and Engineering, Dalian Jiaotong University, Dalian, 116028 China; 4 School of Materials Science and Engineering, University of Science and Technology Beijing, Beijing, 100083 China

**Keywords:** Engineering, Materials science

## Abstract

Carbon steel is strong primarily because of carbides with the most well-known one being θ-Fe_3_C type cementite. However, the formation mechanism of cementite remains unclear. In this study, a new metastable carbide formation mechanism was proposed as ω-Fe_3_C → ω′-Fe_3_C → θ′-Fe_3_C based on the transmission electron microscopy (TEM) observation. Results shown that in quenched high-carbon binary alloys, hexagonal ω-Fe_3_C fine particles are distributed in the martensite twinning boundary alone, while two metastable carbides (ω′ and θ′) coexist in the quenched pearlite. These two carbides both possess orthorhombic crystal structure with different lattice parameters (*a*_θ′_ = *a*_ω′_ = *a*_ω_ = $\sqrt{2}$*a*_α-Fe_ = 4.033 Å, *b*_θ′_ = 2 × *b*_ω′_ = 2 × *c*_ω_ = $\sqrt{3}$*a*_α-Fe_ = 4.94 Å, and *c*_θ′_ = *c*_ω′_ = $\sqrt{3}$*a*_ω_ = 6.986 Å for *a*_α-Fe_ = 2.852 Å). The θ′ unit cell can be constructed simply by merging two ω′ unit cells together along its *b*_ω′_ axis. Thus, the θ′ unit cell contains 12 Fe atoms and 4 C atoms, which in turn matches the composition and atomic number of the θ-Fe_3_C cementite unit cell. The proposed theory in combination with experimental results gives a new insight into the carbide formation mechanism in Fe-C martensite.

## Introduction

The main phase constituents in carbon steels are ferrites (α-Fe) and carbides according to the equilibrium binary Fe-C phase diagram. Therefore carbides have long been considered as a critical phase in strengthening carbon steels. Among all the carbides, the most well-known one is θ-type Fe_3_C cementite, which possesses orthorhombic crystal structure (space group *Pnma*) with its lattice parameter being *a*_θ_ = 4.524 Å, *b*_θ_ = 5.088 Å and *c*_θ_ = 6.741 Å^1,2^. Although the θ-Fe_3_C cementite has been studied extensively due to its importance and popularity in carbon steels^3–16^, its formation mechanism remains unclear. This is particularly true for the θ-Fe_3_C formation during martensitic transformation. One possible reason for this is its ultra-fine particle size, which makes it difficult for the normal characterization techniques to detect the earlier stage of the carbide formation.

Thus far, several types of carbides, which are thought to be the precursors of cementite, have been investigated^17–25^. However, detailed crystal structural relationship between these carbides has not been explained yet. To explain the formation mechanism of the cementite in martensitic structure, a martensite decomposition mechanism (martensite → ε-Fe carbide → cementite) has been proposed previously during tempering at low temperature around 200 °C^23,26–29^. However, most of the alloys used for studying carbide formation were ternary (such as Fe-Ni-C) alloys or other complex alloy systems, which may complicate the analysis and interpretation of carbide formation mechanism. In order to study the fundamental formation mechanism of cementite, the simple binary Fe-C is more appropriate.

Each unit cell of the θ-Fe_3_C cementite with the formula Fe_3_C contains 12 Fe atoms and 4 C atoms, leading to a ratio of Fe to C being 3^1,2,30^. Interestingly, a recently discovered ω-Fe phase located in the martensite twin boundary has three iron atoms in its unit cell as well^31,32^. If one interstitial carbon atom were to join this ω-Fe unit cell, the product would have the formula ω-Fe_3_C. The possibility that there exists certain relationship between the ω-Fe_3_C and θ-Fe_3_C stimulates the investigation into the possible unknown carbides formed earlier than θ-Fe_3_C cementite in the binary Fe-C system.

Metastable hexagonal ω-Fe_3_C phase particles, which are 1 to 2 nm big in size, distribute only at the body-centered cubic (BCC) {112}<111>-type twinning boundary region in twinned high-carbon Fe-C martensite^33–40^. It was observed by *in-situ* heating transmission electron microscopy (TEM) that these twinning boundary ω-Fe_3_C particles eventually transformed into θ-Fe_3_C carbides^41–44^. However, the ω → θ transition speed is too fast for any details to be recorded. Thus, indirect approach is needed to figure out the formation mechanism of these metastable carbides that might exist in the quenched high carbon Fe-C alloys in which several types of ultra-fine carbides with pearlite-like structures have been observed^45,46^. Furthermore, as mentioned above, it is difficult to characterize the crystal structures of ultra-fine carbides via tilting TEM specimens since their particle size is approximately 1–2 nm. Another difficulty comes from the co-existence of several types of fine carbides in localized region. This situation often causes superimposition of relevant selected area electron diffraction (SAED) patterns from several carbide phases.

Therefore, in this paper, we study the metastable carbides by comparative analysis of theoretical and experimental TEM data. The structural models of new carbides were built based on the hexagonal ω-Fe structure and their electron diffraction patterns were simulated using the commercial software, which is designed to simulate the crystal structure, including electron and X-ray diffraction patterns. Then, the calculated diffraction patterns were compared with the observed experimental results. It was confirmed that new kind of metastable carbide, θ′-Fe(C) with 12 Fe atoms and 4 C or less C atoms in its unit cell, existed in the quenched high carbon Fe-C alloys. This carbide has a quite similar crystal structure and the same chemical composition with that of the well-known cementite (θ-Fe_3_C). Thus, understanding the formation mechanism of the θ′-Fe(C) will help us to explore the nature of θ-Fe_3_C cementite.

## Materials and experiment

A Fe-1.6 C (wt.%) binary ingot was prepared in Ar atmosphere within a high-vacuum induction furnace. The ingot was hot-forged into 20 mm-thick plates. Thin plates (approximately 10 mm × 10 mm × 1.0 mm) were then mechanically cut from the hot-forged plates and austenitized at 1150 °C for 30 mins under flowing Ar atmosphere, followed by quenching in water. TEM specimens were prepared from the water-quenched thin plates. The specimens were mechanically ground, polished, and finally ion-milled at room temperature. An ion-mill device (Fischione Model 1050 TEM Mill) was used to prepare the specimens at 4 kV. Sample microstructure was observed using a JEM 2000FX TEM operated at 200 kV. Electron diffraction patterns were calculated using the commercial CrystalMaker software. All electron diffraction patterns shown in the present work were calculated such that the spot intensity saturation was 100 in the software.

## Results and discussion

**Figure 1:**
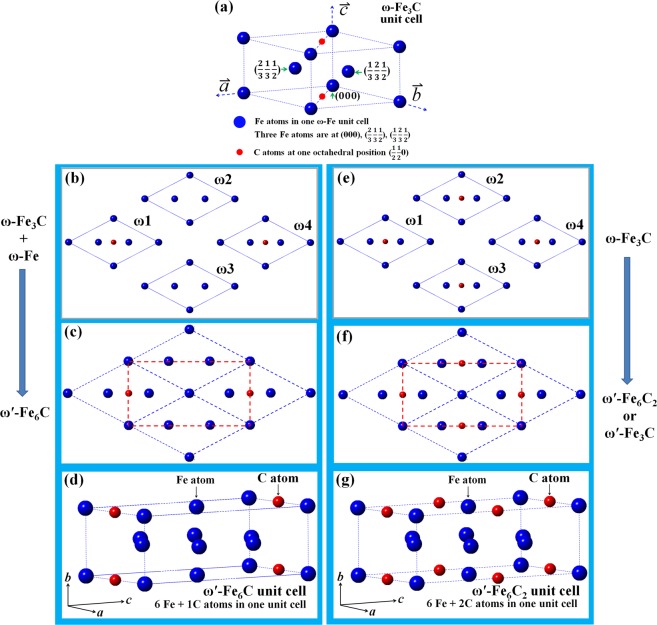
Atomic structure of various carbides. (**a**) Unit cell of ω-Fe_3_C crystal structure. **(b**) Two ω-Fe_3_C and two ω-Fe unit cells projected along their *c* axes. (**c**) Coarsening of the four unit cells of the ω-Fe_3_C and ω-Fe in (**b**) results in the formation of new carbide (ω′-Fe_6_C) outlined by red dashed lines. (**d**) The ω′-Fe_6_C unit cell can have an orthorhombic structure and lattice parameters (*a*_ω′_ = 4.033 Å, *b*_ω′_ = 2.47 Å, and *c*_ω′_ = 6.986 Å for *a*_α-Fe_ = 2.852 Å), and C atom at (0.5 0 0). (**e**) Four ω-Fe_3_C unit cells. (**f**) Coarsening of the four ω-Fe_3_C unit cell in (**e**) results in the formation of a new carbide (ω′-Fe_6_C_2_ or ω′-Fe_3_C) with the same crystal structure and lattice parameters as the ω′-Fe_6_C. (**g**) The ω′-Fe_6_C_2_ atomic structure in one unit cell.

TEM observations revealed that ultra-fine ω-Fe_3_C particles exist at twinning boundary region in twinned Fe-C martensite, and the ω-Fe_3_C has a hexagonal crystal structure with lattice parameters of a = *a*_ω_ = $\sqrt{2}$*a*_α-Fe_ = 4.033 Å, *c*_ω_ = 1/2 × $\sqrt{3}$*a*_α-Fe_ = 2.47 Å for *a*_α-Fe_ = 2.852 Å^31,33,35–38^. The ω-Fe_3_C unit cell structure can be seen from Fig. 1(a).

### ω′-variants

**Figure 2:**
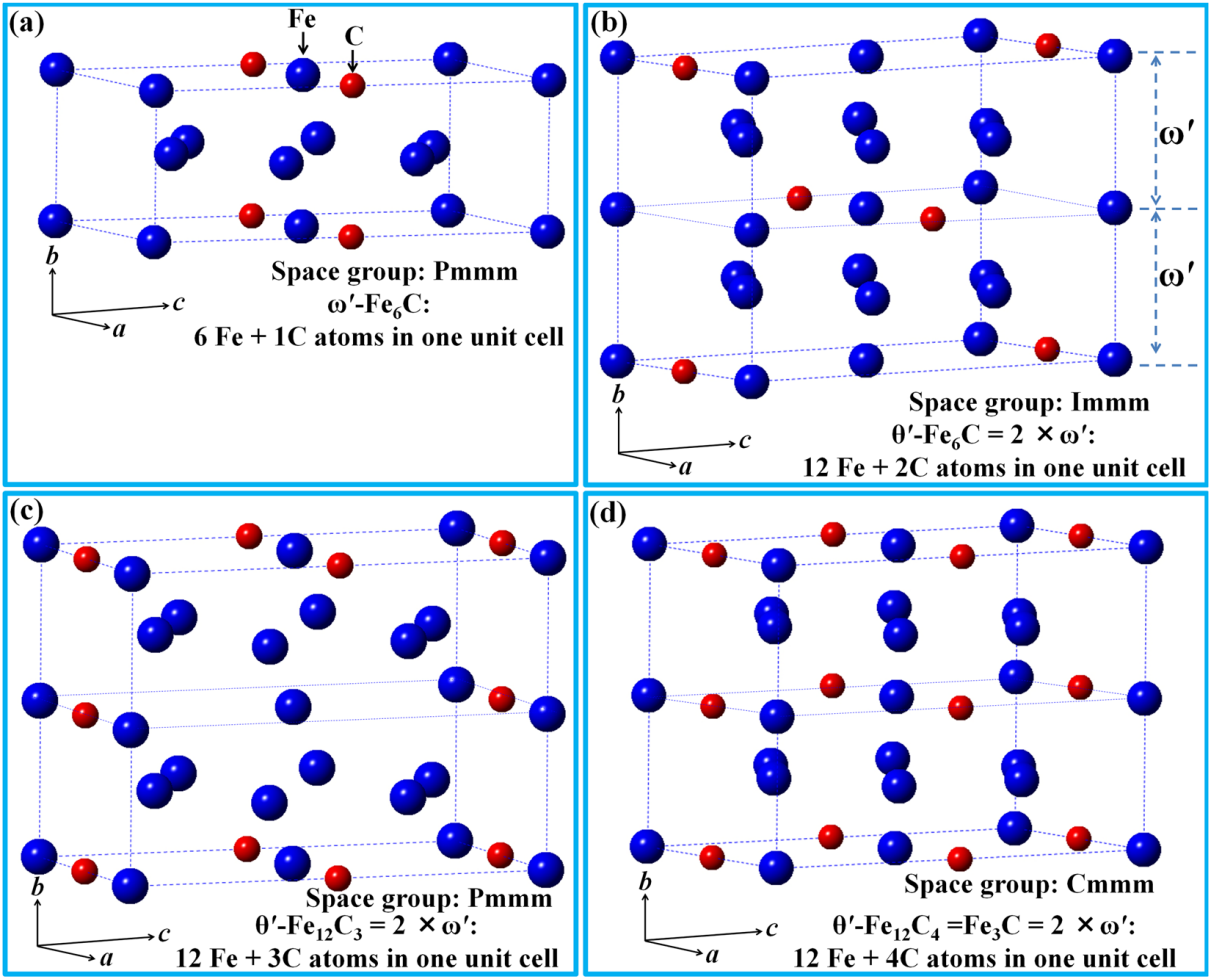
Atomic structure of various carbides. (**a**) Unit cell of one ω′-Fe_6_C variant with one C atom at (0 0 0.5). (**b**) New θ′ variant (θ′-Fe_12_C_2_ or θ′-Fe_6_C) formed by merging the ω′-Fe_6_C variant with one C atom at (0.5 0 0) (Fig. 1(d)) and the ω′-Fe_6_C variant with one C atom at (0 0 0.5) in (**a**) along *b* axis. (**c**) New θ′ (θ′-Fe_12_C_3_ or θ′-Fe_4_C) variant. (**d**) New θ′ variant of θ′-Fe_12_C_4_ or θ′-Fe_3_C formed by doubling the ω′-Fe_6_C_2_ in Fig. 1(f) along *b* axis. All θ′ have an orthorhombic unit cell with lattice parameters of *a*_θ′_ = 4.033 Å, *b*_θ′_ = 2 × 2.47 Å = 4.94 Å, and *c*_θ′_ = 6.986 Å for *a*_α-Fe_ = 2.852 Å).

As an interstitial atom, the position of carbon atoms in crystals determines carbide structure. Two different coarsening behaviors of the ultra-fine ω-Fe_3_C particles are illustrated in Fig. 1. Figure 1(a) shows the atomic structure of one ω-Fe_3_C unit cell. The coarsening route (Fig. 1(b)) will generate a new kind of carbide, with its unit cell outlined by red dashed lines in Fig. 1(c). Its corresponding three-dimensional (3D) atomic structure is shown in Fig. 1(d). There are six iron atoms and one carbon interstitial atom in this unit cell, which has been designated as ω′-Fe_6_C in our previous study^46^. On the other hand, if the positions of two ω-Fe_3_C (ω1, ω4) and two ω-Fe (ω2, ω3) in Fig. 1b exchange, the ω′-Fe_6_C will has the carbon atom at (0 0 0.5) as shown in Fig. 2a. Obviously, ω′-Fe_6_C has two forms because of the different carbon atom position as shown in Figs. 1d and 2a.

When the coarsening of the ultra-fine ω-Fe_3_C particles follows the route shown in Fig. 1(e–g), new carbide consisting of six iron atoms and two carbon atoms in its unit cell will form as shown in Fig. 1(g), with its formula being ω′-Fe_6_C_2_ or ω′-Fe_3_C. There is no any difference in the calculated electron diffraction patterns between the ω′-Fe_6_C_2_ and ω-Fe_3_C carbides since both carbide crystals have the exact same atomic positions. As can be seen in Fig. 1, the transformation of the ω-Fe_3_C hexagonal structure to an orthorhombic structure depends on the carbon content and/or positons alone. Once the ordering of carbon atoms occurs, the orthorhombic structure can form in a spontaneous way. The electron diffraction spots associated with such an ordering has been observed in the ω′-Fe_6_C carbide^46^.

Since there is no obvious difference in the calculated electron diffraction patterns between ω-Fe_3_C carbide and ω-Fe phase^45^, the three phases (ω-Fe, ω-Fe_3_C, ω′-Fe_6_C_2_ (ω′-Fe_3_C)) would have similar electron diffraction patterns, which may cause difficulty in charactering the carbides experimentally. Simply speaking, there are three phases (hexagonal ω-Fe, orthorhombic ω-Fe_3_C and ω′-Fe_6_C_2_ (ω′-Fe_3_C)) present theoretically. However, it is difficult to distinguish among them since they show similar electron diffraction pattern experimentally. Formation of this new ω′-Fe_3_C carbide can actually explain why the ultra-fine ω-Fe_3_C particles never grow big in real materials. The ω-Fe_3_C particle size is just only 1–2 nm.

The carbide coarsening can be achieved via the several fine ω-Fe_3_C particles merged together. The driving force for the movement of fine ω-Fe_3_C particles comes from the recrystallization of ultra-fine α-Fe matrix grains. There are two crystalline phases, namely fine α-Fe as a matrix grain and fine ω-Fe_3_C particles at the α-Fe twinning boundaries that co-exist in the twinned martensitic structure. Thus, the coarsening behavior is actually controlled by the recrystallization process of the α-Fe matrix grains upon tempering. The α-Fe recrystallization results in a movement of the α-Fe grain boundaries and/or twinning boundaries, which promotes the ω-Fe_3_C particles at the boundaries to move and meet other ω-Fe_3_C particles. The coarsening behavior of the fine ω-Fe_3_C particles and the recrystallization process of the ultra-fine α-Fe grains have been experimentally confirmed and explained in our previous work^35,40,42–44^.

### θ′-variants

Following the same coarsening mechanism explained in Fig. 1, new carbide, here designated as θ′, can be formed by combining two variants of ω′. The atomic structures of possible θ′ variants are shown in Fig. 2. Figure 2(a) shows one of the ω′ variants, while the other two ω′ variants are shown in Fig. 1(d,g). After two variants of ω′ merge together along its *b* axis, three θ′ variants (θ′-Fe_12_C_2_ or θ′-Fe_6_C (Fig. 2(b)), θ′-Fe_12_C_3_ or θ′-Fe_4_C (Fig. 2(c)), θ′-Fe_12_C_4_ or θ′-Fe_3_C (Fig. 2(d))) can be formed. Thus, the θ′ carbides possess lattice parameter of *a*_θ′_ = 4.033 Å, *b*_θ′_ = 2 × 2.47 Å = 4.94 Å, and *c*_θ′_ = 6.986 Å and retain an orthorhombic crystal structure. During the coarsening of fine ω′ particles, one ω′ particle with the crystal structure in Fig. 1(g) may combine with another ω′ particle with the same crystal structure along its *b*_ω′_ axis. When this occurs, it is possible for a θ′-Fe_3_C carbide particle to form.

The formation of θ′-Fe_12_C_4_ or θ′-Fe_3_C variant involves merging two ω′-Fe_6_C_2_ or ω′-Fe_3_C carbide particles together alone without any atomic movement or variation in carbon content. It can be seen from Figs. 1 and 2 that the position of both Fe and C atoms during the ω → ω′ → θ′ transition are kept unchanged, meaning that this transition depends completely on the size of the ω-Fe_3_C carbide particle. The atomic positions of Fe and C atoms in the ω-Fe_3_C, ω′-Fe_3_C and θ′-Fe_3_C unit cells have been listed in the Tables 1–3, respectively.

**Table 1:** The fraction coordination of three Fe atoms and one C atom of ω-Fe_3_C in hexagonal structure with the lattice parameters of *a*_ω_ = $\sqrt{2}$*a*_α-Fe_ = 4.033 Å_,_ *c*_ω =_ $\frac{\sqrt{3}}{2}$*a*_α-Fe_ = 2_._47 Å for *a*_α-Fe_ = 2.852 Å.

Atoms	*a*_ω_	*b*_ω_	*c*_ω_
Fe1	0	0	0
Fe2	2/3	1/3	1/2
Fe3	1/3	2/3	1/2
C	1/2	1/2	0

**Table 2:** The fraction coordination of six Fe atoms and two C atom of ω′-Fe_3_C in orthorhombic structure with the lattice parameters of *a*_ω′_ = $\sqrt{2}$*a*_α-Fe_ = 4.033 Å_,_ *b*_ω′ =_ $\frac{\sqrt{3}}{2}$*a*_α-Fe =_ 2.47 _Å_, *c*_ω′_ = $\sqrt{6}$*a*_α-Fe_ = 6.986 Å for *a*_α-Fe_ = 2.852 Å. New variant of the ω′ carbide can be formed depending on the carbon concentration and positions.

Atoms	*a*_ω′_	*b*_ω′_	*c*_ω′_
Fe1	0	0	0
Fe2	1/2	0	1/2
Fe3	1/2	1/2	1/6
Fe4	1/2	1/2	5/6
Fe5	0	1/2	1/3
Fe6	0	1/2	2/3
C1	1/2	0	0
C2	0	0	1/2

**Table 3:** The fraction coordination of twelve Fe atoms and four C atom of θ′-Fe_3_C in orthorhombic structure with the lattice parameters of *a*_θ′_ = $\sqrt{2}$*a*_α-Fe_ = 4.033 Å_,_ *b*_θ′ =_ $\sqrt{3}$*a*_α-Fe_ = 4.94 _Å_, *c*_θ′_ = $\sqrt{6}$*a*_α-Fe_ = 6.986 Å for *a*_α-Fe_ = 2.852 Å. Various variants are formed depending on the position and concentration of the intestinal carbon atoms.

Atoms	*a*_θ′_	*b*_θ′_	*c*_θ′_
Fe1	0	0	0
Fe2	1/2	0	1/2
Fe3	0	1/2	0
Fe4	1/2	1/2	1/2
Fe5	1/2	1/4	1/6
Fe6	1/2	1/4	5/6
Fe7	0	1/4	1/3
Fe8	0	1/4	2/3
Fe9	1/2	3/4	1/6
Fe10	1/2	3/4	5/6
Fe11	0	3/4	1/3
Fe12	0	3/4	2/3
C1	0	0	1/2
C2	1/2	0	0
C3	0	1/2	1/2
C4	1/2	1/2	0

**Figure 3:**
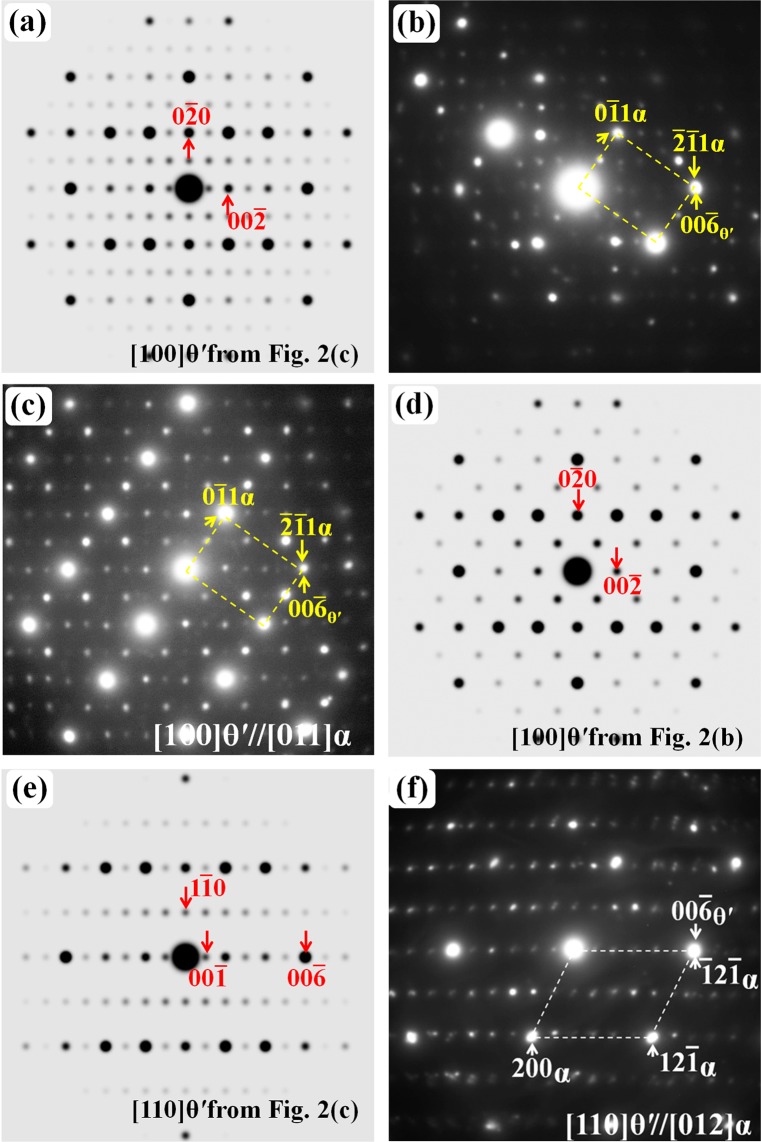
Electron diffraction patterns of the θ′ variants: (**a**) Simulated [100] zone axis pattern of the θ′-Fe_12_C_3_ carbide. (**b**) Experimental pattern consisting of the diffraction spots from the [011] α-Fe zone axis and [100] θ′-Fe_12_C_3_ carbide. (**c**) Experimental pattern consisting of three sets of diffraction spots: [011] α-Fe zone axis, [100] θ′-Fe_12_C_3_ carbide and [100] zone axis of [100] θ′-Fe_12_C_2_ carbide. (**d**) Simulated [100] zone axis pattern of the θ′-Fe_12_C_2_ carbide. (**e**) Simulated [110] zone axis pattern of the θ′-Fe_12_C_3_ carbide. (**f**) Experimental electron diffraction patterns consisting of the spots from the [012] α-Fe zone axis and [110] θ′-Fe_12_C_3_ carbide.

As explained in Figs. 1 and 2, ω′ and θ′ can have other variants with lower carbon content than that in the ω′-Fe_6_C_2_ (ω′-Fe_3_C) and θ′-Fe_12_C_4_ (θ′-Fe_3_C) unit cells. The ω′ variant (ω′-Fe_6_C) has been experimentally observed previously.^46^ Fig. 3 shows the evidence that there exist other type θ′ variants in the quenched high carbon Fe-C alloys. Simulated electron diffraction pattern of the θ′-Fe_12_C_3_ carbide with its [100] zone axis parallel to the electron beam is shown in Fig. 3(a), while the corresponding experimental electron diffraction pattern is shown in Fig. 3(b). The experimental diffraction pattern is composed of two sets of diffraction spots. One set is from [011] α-Fe zone axis and the other is from the [100] zone axis of the θ′-Fe_12_C_3_ carbide as shown in Fig. 3(a). The mixed electron diffraction patterns of α-Fe and fine carbides are frequently observed in the quenched Fe-C alloy with pearlite structure since both phases have ultra-fine particles (the region selected for experimental observations depends on the selected aperture size in TEM equipment, the smallest diameter size of the aperture is about 250 nm).

Figure 3(c) shows a particular experimental pattern, which is composed of three sets of diffraction spots: (1) the strong spots from [011] α-Fe zone axis as outlined by the yellow dashed lines, (2) the spots shown in Fig. 3(a), which is from [100] zone axis of the θ′-Fe_12_C_3_ carbide, and (3) the spots [100] zone axis of the θ′-Fe_12_C_2_ carbide as shown in Fig. 3(d). Experimental diffraction patterns are usually obtained from an area of several hundreds of nanometers in diameter. Thus, the diffraction pattern is composed of several sets of diffraction spots, which come from various carbides present in the pearlite-like region. Figure 3(e) shows the simulated electron diffraction patterns of the θ′-Fe_12_C_3_ carbide along its [110] zone axis. This pattern can also be observed experimentally along the α-Fe [012] zone axis as shown in Fig. 3(f).

The results in Fig. 3 reveal that various θ′ variants can co-exist in the quenched sample, and the formation of different type of variants is dependent on carbon concentration and positions. The possible variants of both θ′ carbides and ω′ carbides are summarized and listed in Table 4. Both ω′ and θ′ carbides possess orthorhombic crystal structure. The unit cell of θ′ carbides is composed of two ω′ unit cells merged along its *b* axis. The formation mechanism of θ′ carbides is the variation in carbon atoms or concentration on different atomic planes, which causes an ordering structure of ω-Fe. Since the carbon atoms or concentration are the same in (001) planes of ω′-Fe_6_C_2_ (ω′-Fe_3_C) and θ′-Fe_12_C_4_ (θ′-Fe_3_C) and the electron diffraction patterns of ω-Fe_3_C, ω′-Fe_6_C_2_ (ω′-Fe_3_C) and θ′-Fe_12_C_4_ (θ′-Fe_3_C) are similar, no carbon-ordering diffraction spots could be observed. However, that is not to say the ω′-Fe_6_C_2_ (ω′-Fe_3_C) or θ′-Fe_12_C_4_ (θ′-Fe_3_C) does not exist in the sample.

**Table 4:** The structural parameters and chemical composition of possible variants of the ω′ and θ′ carbides. The electron diffraction patterns of the ω′-Fe_6_C_2_ (ω′-Fe_3_C) and θ′-Fe_12_C_4_ (θ′-Fe_3_C) are the same with that of the ω-Fe_3_C.

Metastable carbide	Variant composition	Notes
ω′, orthorhombic *a*_ω′_ = 4.033 Å, *b*_ω′_= 2.47 Å, *c*_ω′_ = 6.986 Å	ω′-Fe_6_C	C atoms at different (001) atomic planes
	ω′-Fe_6_C	
	ω′-Fe_6_C_2_ (ω′-Fe_3_C)	
θ′, orthorhombic *a*_θ′_ = 4.033 Å, *b*_θ′_= 4.94 Å, *c*_θ′_ = 6.986 Å	θ′-Fe_12_C_2_ (θ′-Fe_6_C)	C concentration varies at different (001) atomic planes
	θ′-Fe_12_C_3_ (θ′-Fe_4_C)	
	θ′-Fe_12_C_4_ (θ′-Fe_3_C)	

### θ-Fe_3_C cementite

**Figure 4:**
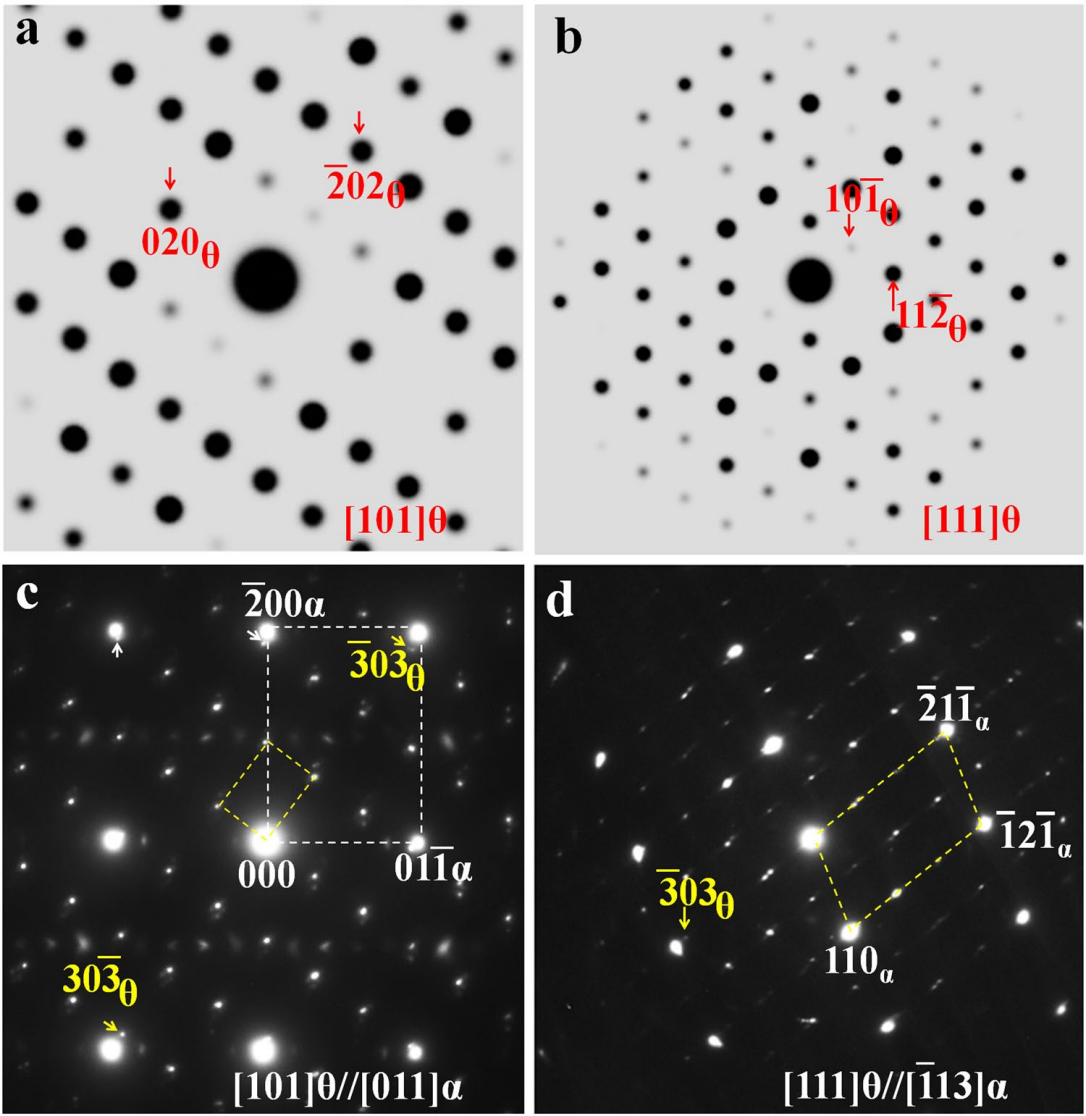
Simulated θ-Fe_3_C electron diffraction patterns: (**a**) [101]θ and (**b**) [111]θ. The corresponding experimental patterns observed along the zone axes of (**c**) [011]α and (**d**) [113]α.

The diffraction patterns of θ-Fe_3_C cementite from two different zone axes ([101]_θ_ in Fig. 4(a) and [111]_θ_ in Fig. 4(b)) are shown here in comparison with that of previous carbides (ω-Fe_3_C, ω′-Fe_6_C_2_ (ω′-Fe_3_C) or ω′-Fe_6_C and various θ′-variants). It can be seen from Fig. 4(c), the experimental $\bar{3}$03_θ_ spot is completely separated from the α-Fe $\bar{2}$1 $\bar{1}$ spot, unlike the corresponding ω and ω′ or θ′ spots, which overlap perfectly with the corresponding α-Fe spots. This kind of separation can also be clearly observed in other direction as shown in Fig. 4(d). The results shown in Fig. 4 explain that the carbide with the well-known cementite structure has lost the perfect overlapping in diffraction spots compared with other carbides mentioned earlier.

**Figure 5:**
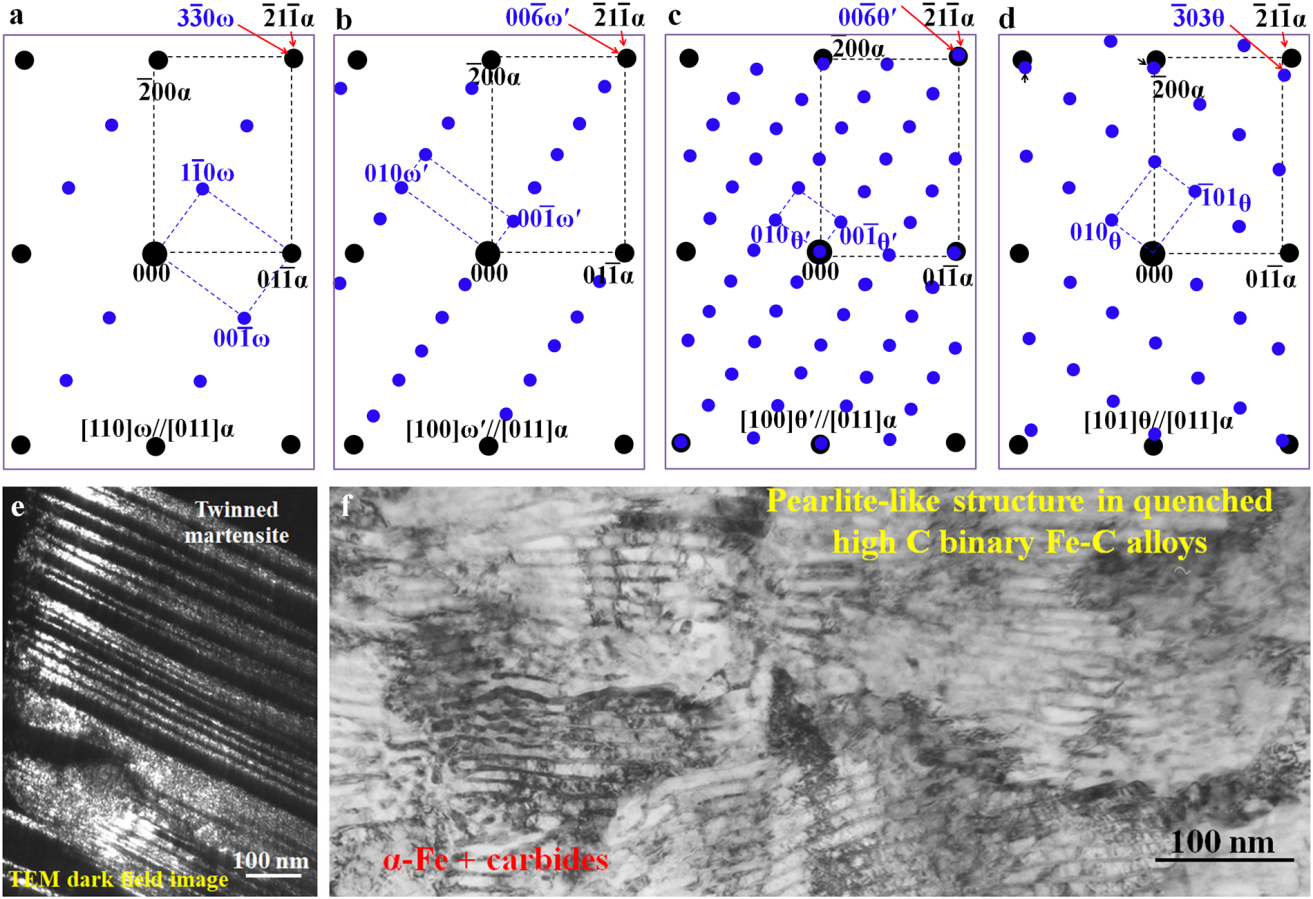
Schematic electron diffraction patterns between α-Fe and the (**a**) ω, (**b**) ω′, (**c**) θ′, and (**d**) the θ-Fe_3_C carbides. All patterns are along the [011]_α_ zone axis.(**e**) Dark field TEM image revealing the twinned structure, which corresponds to the ω existing region. (**f**) TEM bright field image showing the pearlite-like structure corresponding to the existing region of the ω′, θ′, and θ fine carbide region.

The electron diffraction patterns of ω-related carbide structures (ω, ω′, θ′ and θ) in quenched high-carbon binary Fe-C alloys are illustrated in Fig. 5. Figure 5(a–d) show the schematics of diffraction patterns based on the experimental results.^45,46^ All these patterns are obtained from the same α-Fe [011] zone axis. The pattern shown in Fig. 5(a) can only be observed within twinned martensite (Fig. 5(e)). The pattern (Fig. 5(a)) reveals a complete overlapping between the 211 α and the 330 ω spots. In Fig. 5(b), the ω-Fe_3_C diffraction pattern is converted into ω′-Fe_6_C with an ordering pattern and the original three spots (1 $\bar{1}$0_ω_, 2 $\bar{2}$0_ω_ and 3 $\bar{3}$0_ω_) turn out to be six spots between the transmitted (central) and the 211 α diffraction spots. When two ω′-Fe_6_C unit cells merge together to form a θ′-Fe_12_C_3_ variant with its b_θ′_ = 2 bω_′_, an extra row of diffraction spots would occur in reciprocal space as shown in Fig. 5(c). The corresponding diffraction pattern from θ-Fe_3_C is shown in Fig. 5(d) for a comparison with that of the ω-Fe_3_C-related carbides to show a crystal structural similarity among these carbides. The patterns shown in Fig. 5(b–d) are normally observed in quenched pearlite-like microstructure like that shown in Fig. 5(f). Not only can ω′ carbides be observed in the pearlite-like microstructure, but θ′ and θ fine carbides can also be observed in the same pearlite-like region. Nevertheless, it is difficult to differentiate these carbides based on particle size or morphology alone since all of them are several nanometers in size.

In experimental TEM observations, these three carbides (ω, ω′ and θ′) can be identified easily based on the superimposition of certain diffraction spots on 211 α-Fe. Once these carbides start to transform into the well-known θ-Fe_3_C cementite, the separation of the 303 θ diffraction spots from 211 α-Fe spots can be clearly seen as shown in Fig. 5(d). Such a separation will produce complex diffraction patterns and cause difficulty in carbide characterization.

## Conclusion

Ultra-fine carbides formed in quenched Fe-C alloys were investigated by comparing experimental results with simulated electron diffraction patterns.

1. Based on the unit cells of the ω′-Fe_3_C and its variants, an orthorhombic θ′ carbide structure with lattice parameter of *a* = 4.033 Å, *b* = 4.94 Å, and *c* = 6.986 Å was constructed and experimentally confirmed. The θ′ carbide can be: θ′-Fe_12_C_2_ (or θ′-Fe_6_C), θ′-Fe_12_C_3_ (or θ′-Fe_4_C) and θ′-Fe_12_C_4_ (or θ′-Fe_3_C) compounds.
2. A transition route (ω → ω′→ θ′) has been proposed during the coarsening of ultra-fine ω-Fe_3_C particles to explain the formation mechanism of the θ′ carbide with various variants. The transition occurs accompanying the variation in the position and concentration of carbon atoms, while the position of Fe atoms is kept unchanged.
3. It was observed that the ω′, θ′ and θ metastable carbides with ultra-fine particle size co-existed in the pearlite-like microstructure of quenched high carbon Fe-C alloys.
